# Application of regularized regression to identify novel predictors of mortality in a cohort of hemodialysis patients

**DOI:** 10.1038/s41598-021-88655-0

**Published:** 2021-04-29

**Authors:** Stanislas Werfel, Georg Lorenz, Bernhard Haller, Roman Günthner, Julia Matschkal, Matthias C. Braunisch, Carolin Schaller, Peter Gundel, Stephan Kemmner, Salim S. Hayek, Christian Nusshag, Jochen Reiser, Philipp Moog, Uwe Heemann, Christoph Schmaderer

**Affiliations:** 1 Department of Nephrology, Klinikum rechts der Isar, Technical University Munich, Ismaninger Str. 22, 81675 Munich, Germany; 2 Institute of Medical Informatics, Statistics and Epidemiology, Technical University Munich, Munich, Germany; 3 Transplant Center, University Hospital Munich, Ludwig-Maximilians-University (LMU), Munich, Germany; 4 Division of Cardiology, Department of Medicine, University of Michigan, Ann Arbor, MI USA; 5 Department of Medicine, Rush University Medical Center, Chicago, IL USA; 6 Departement of Nephrology, University Hospital Heidelberg, Heidelberg, Germany

**Keywords:** Nephrology, Renal replacement therapy, Data integration

## Abstract

Cohort studies often provide a large array of data on study participants. The techniques of statistical learning can allow an efficient way to analyze large datasets in order to uncover previously unknown, clinically relevant predictors of morbidity or mortality. We applied a combination of elastic net penalized Cox regression and stability selection with the aim of identifying novel predictors of mortality in a cohort of prevalent hemodialysis patients. In our analysis we included 475 patients from the “rISk strAtification in end-stage Renal disease” (ISAR) study, who we split into derivation and confirmation cohorts. A wide array of examinations was available for study participants, resulting in over a hundred potential predictors. In the selection approach many of the well established predictors were retrieved in the derivation cohort. Additionally, the serum levels of IL-12p70 and AST were selected as mortality predictors and confirmed in the withheld subgroup. High IL-12p70 levels were specifically prognostic of infection-related mortality. In summary, we demonstrate an approach how statistical learning can be applied to a cohort study to derive novel hypotheses in a data-driven way. Our results suggest a novel role of IL-12p70 in infection-related mortality, while AST is a promising additional biomarker in patients undergoing hemodialysis.

## Introduction

End-stage renal disease (ESRD) with hemodialysis treatment continues to be associated with dramatically increased morbidity and mortality. While cardiovascular mortality represents the prevalent cause of death in these patients, death due to other causes such as infection is also more frequent than in the general population^1^. Several risk factors of cardiovascular and all-cause mortality have been identified and risk scores have been successfully established based on these^2–7^. Beyond the quantification of a personal prognosis, the identification of novel risk factors can lead to hypothesis generation for further research, novel therapeutic interventions and finally lead to improvements in patient outcomes.

Machine learning approaches for survival analysis can allow efficient selection of the most relevant risk factors even when a large number of potential predictors for only a relatively small patient population is available^8,9^. Among them, Cox regression using “elastic net” regularization allows selection even in settings where there are more predictors than events in the sample^10^. Here regularization refers to the procedure of penalizing and thereby shrinking the regression coefficients while (in the case of “elastic net”) allowing them to become zero and therefore omitting variables with less predictive power for the outcome. An advantage of this procedure compared to many other machine learning techniques is that it produces a model which is accessible to interpretation by clinician scientists, in a similar way as common multivariable Cox regression. This allows to draw conclusions from the resulting model itself such as e.g. generation of hypotheses for future therapeutic interventions.

A more recent advancement is a combination of regularization with resampling, termed “stability selection”, which consists of recalculating the regularized regression many times with random subgroups of the available patient population in order to select the variables which show most consistent association with the outcome. This reportedly results in a more reliable selection in settings with a large number of potential predictors compared to the number of patients^11^.

Here we report on the application of elastic net regularization combined with stability selection for the identification of relevant predictors of mortality in the “ISAR” cohort of prevalent hemodialysis patients^12^.

## Results

### Characteristics of the study population and collected data

**Figure 1:**
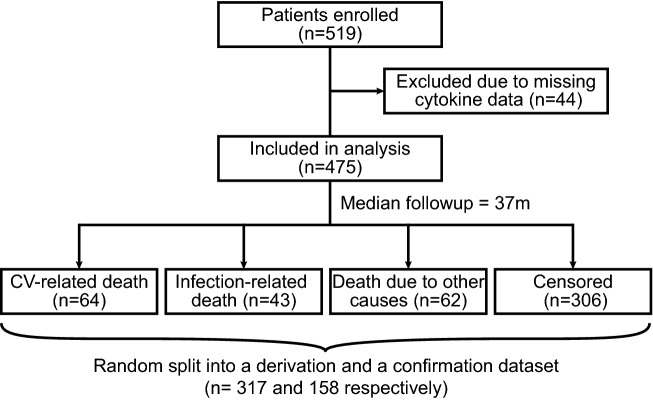
Patient flow diagram for the ISAR dialysis trial. The censoring for patients without an event occurred largely (≈ 80%) at the time point of last follow-up (4 years after study initiation). Death due to other causes also included unknown cause of death. Further details of the derivation and confirmation datasets are presented in Table 1. CV, cardiovascular.

The overall study design and rationale of the ISAR cohort trial have been described in our previous report^12^. Briefly summarized, prevalent hemodialysis patients were recruited in 17 centers in and around Munich, Germany and followed up for a median time of 37 months (interquartile range of 25–49 months). A total of 475 patients were included in the analysis (Fig. 1). Baseline characteristics for this population were previously reported^5^. Mortality events were classified into three categories: cardiovascular, infectious/sepsis and other (or unknown), with 64, 43 and 62 respective events occurring during the follow-up period (Fig. 1). Overall over 100 possible predictors were collected for the patients in the study population including clinical parameters, comorbidities and medications, blood value measurements and static retinal vessel analyses (Table S1).

We randomly separated the total cohort into a derivation and confirmation dataset (n = 317 and 158 respectively) with similar characteristics (Fig. 1). The sets were specifically similar with regard to age, gender, dialysis patient adapted Charlson comorbidity index (CCI)^13^, serum IL-6 and mortality (Table 1).

**Table 1:** Matching characteristics of the derivation and confirmation datasets.

	Total	Derivation	Confirmation	p-value
n (%total)	475 (100%)	317 (67%)	158 (33%)	
Age (year)	68 [55–77]	67 [55–77]	69 [54–77]	0.72
Sex (%male)	331 (70%)	219 (69%)	112 (71%)	0.77
Comorbidity index	3 [1–6]	3 [2–6]	4 [1–7]	0.96
Serum IL-6 (pg/ml)	9.4 [5.6–16.1]	9.2 [5.6–15.6]	10.2 [5.7–16.6]	0.58
All-cause mortality	169 (36%)	112 (35%)	57 (36%)	0.95
Cardiovascular mortality	64 (13%)	44 (14%)	20 (13%)	0.82
Mortality D/T infection	43 (9%)	27 (9%)	16 (10%)	0.68
Mortality D/T other causes	62 (13%)	41 (13%)	21 (13%)	1.00
1 year mortality	35 (7%)	24 (8%)	11 (7%)	0.96

Nominal variables are reported as counts and percentages, ordinal/continuous variables as median and interquartile range. P-values were calculated using a Chi-squared and a Mann–Whitney test respectively. Dialysis-patient adapted comorbidity index was calculated as described by Liu et al.^13^.

D/T, due to.

### Application of a combination of elastic net regularization and stability selection for the identification of mortality predictors

**Figure 2:**
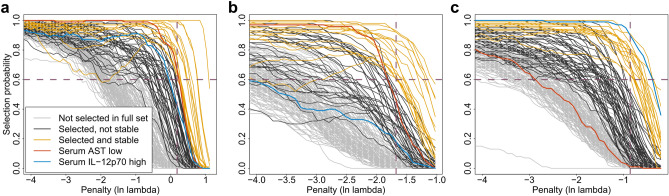
Stability paths of the elastic net regression for all-cause (**a**), cardiovascular (**b**), and infection-associated mortality (**c**). Each curve represents either a nominal predictor or a group (top/bottom quintile) of an ordinal/continuous predictor. The colors are coded as indicated. Vertical dashed lines represent the penalty parameter (ln-lambda) chosen by the stability selection, horizontal dashed lines represent the predefined selection threshold of 60%. Variables above this threshold at the selected penalty were considered stable. "High" and "low" indicates that the value falls within the top or bottom quintile of the total study population (see “Methods”).

Variable selection with consecutive stability selection in the derivation dataset resulted in a substantially reduced predictor subset compared to using only the elastic net regression (Figure S1 and Fig. 2). Most of the predictors passing selection criteria for all-cause mortality or the cause-specific events showed significant associations with mortality in the withheld confirmation cohort (Table 2). Among the comorbidities diabetes mellitus, pulmonary hypertension, atrial fibrillation (AF), history of myocardial infarction, neoplasia, atherosclerosis (other than CHD) and peripheral artery disease (PAD) were selected and confirmed. Most of these have in fact previously been shown to have a significant effect on mortality^13^. Other previously reported predictors were catheter use for dialysis, blood pressure, hemoglobin levels, serum creatinine, CRP^2,14^, use of oral anticoagulation^15^, serum levels of IL-6 and YKL-40, as well as IP-10^5^. We could also observe a detrimental effect of low serum triglycerides in our population as has been reported previously^16^. Interestingly, we also found low levels of AST to be associated with reduced all-cause mortality, while high levels of IL-12p70 were associated with an increased infection-associated mortality (Table 2). The association of AST with mortality is a relatively novel finding in dialysis patients^17^. The positive association of IL-12p70 with mortality in these patients is to our knowledge a novel finding. We therefore further analyzed these predictors in our study population.

**Table 2:** Summary of the stably selected variables and their confirmation.

	Derivation						Confirmation									n
	Stable selection			Sign of EN coefficient			All-cause			Cardiovascular			Infection-associated			
	AC	CV	INF	AC	CV	INF	HR	CI (95%)	p-value	HR	CI (95%)	p-value	HR	CI (95%)	p-value	
Age (per 10 years)	x	x	x	+	+	+	1.64	(1.31–2.06)	< 0.001*	1.86	(1.23–2.81)	0.003*	1.75	(1.12–2.73)	0.014*	158
Atherosclerosis (non CHD)	x		x	+		+	2.14	(1.26–3.61)	0.005*				2.70	(1.01–7.21)	0.048*	47
Atrial fibrillation	x			+			2.67	(1.58–4.52)	< 0.001*							42
CVD		x			+					2.35	(0.97–5.71)	0.059^#^				62
Dialysis catheter	x	x	x	+	+	+	3.72	(1.59–8.72)	0.002*	3.47	(0.80–15.12)	0.098^#^	4.09	(0.92–18.12)	0.064^#^	8
DM	x	x		+	+		2.00	(1.19–3.37)	0.009*	1.38	(0.57–3.35)	0.470				61
H.o. amputation due to PAD	x	x	x	+	+	+	1.57	(0.67–3.67)	0.296	2.43	(0.71–8.35)	0.158	0.90	(0.12–6.81)	0.918	10
H.o. MI		x			+					2.49	(0.97–6.40)	0.057^#^				37
H.o. neoplasia		x			+					2.22	(0.90–5.46)	0.082^#^				42
HF	x			+			2.57	(1.49–4.41)	0.001*							35
HRD (non AF)			x			+							2.07	(0.59–7.31)	0.256	19
Oral anticoagulation	x			+			3.10	(1.71–5.64)	< 0.001*							22
Other ac (dialysis)			x			+							0.00	(0.00–Inf)	0.998	7
PAD	x		x	+		+	2.13	(1.23–3.69)	0.007*				2.56	(0.93–7.05)	0.069^#^	31
Pulmonary hypertension		x			+					3.58	(1.19–10.81)	0.023*				13
DBP (24 h) low			x			+							3.41	(1.18–9.80)	0.023*	20
MAP (24 h) low			x			+							2.22	(0.71–6.87)	0.169	23
Immunosuppression		x			+					0.47	(0.06–3.48)	0.457				17
Kt/V high			x			−							2.85	(0.98–8.26)	0.054	25
HB low			x			+							3.26	(1.18–9.03)	0.023*	29
Serum AST low	x			−			0.31	(0.10–0.98)	0.046*							23
Serum cholesterol high		x			+					0.22	(0.03–1.67)	0.144				24
Serum creatinine high	x			−			0.44	(0.18–1.11)	0.083^#^							30
Serum creatinine low	x	x		+	+		1.34	(0.77–2.33)	0.295	1.77	(0.72–4.35)	0.210				44
Serum hsCRP high	x			+			1.82	(1.00–3.28)	0.048*							31
Serum IFN-gamma high			x			+							0.95	(0.27–3.33)	0.934	29
Serum IL-12p70 high			x			+							3.12	(1.16–8.41)	0.024*	33
Serum IL-13 low			x			+							0.26	(0.03–1.95)	0.190	32
Serum IL-6 high	x	x	x	+	+	+	2.96	(1.73–5.07)	< 0.001*	1.98	(0.75–5.20)	0.165	4.49	(1.67–12.05)	0.003*	36
Serum IP-10 high		x			+					1.19	(0.45–3.10)	0.726				38
Serum IP-10 low			x			−							0.57	(0.13–2.53)	0.462	30
Serum iron low			x			+							1.94	(0.62–6.05)	0.251	26
Serum LDL high		x			+					0.49	(0.11–2.13)	0.343				24
Serum phosphate low		x			+					1.28	(0.49–3.33)	0.615				36
Serum triglycerides low	x	x		+	+		2.30	(1.16–4.56)	0.018*	1.20	(0.28–5.19)	0.809				16
Serum YKL-40 high	x			+			2.51	(1.44–4.35)	0.001*							32

For ordinal variables "high" and "low" indicates that the value falls within the top or bottom quintile of the total study population (see “Methods”).

ac, anticoagulation; AC, all-cause mortality; AF, atrial fibrillation; AST, Aspartate transaminase; CHD, coronary heart disease; CI(95%), 95% confidence interval of the hazard ratio; CV, cardiovascular mortality; CVD, cardiovascular disease; D/T, due to; DBT (24 h), average (24 h) diastolic blood pressure; DM, Diabetes mellitus; EN, elastic net; ESRD, end-stage renal disease; H.o., history of; HB, hemoglobin; HF, heart failure; HR, hazard ratio; HRD, heart rhythm disorder; INF, infection-associated mortality; MAP (24 h), average (24 h) mean arterial pressure; MI, myocardial infarction; n, number of non-zero observations of the respective predictor in the confirmation group; PAD, peripheral artery disease.

*p < 0.05; ^#^, two-sided p < 0.1 and same effect direction as in the elastic-net regression derivation dataset for respective all-cause or cause-specific mortality. Effect direction in the derivation dataset is indicated as sign of elastic net coefficient: + , regularized HR > 1; −, regularized HR < 1. Statistics were calculated in the confirmation dataset using univariate Cox regression. Tests were only performed in the confirmation dataset for predictors which passed stability selection for the respective outcome in the derivation dataset.

### Serum AST as predictor of mortality in dialysis patients

**Figure 3:**
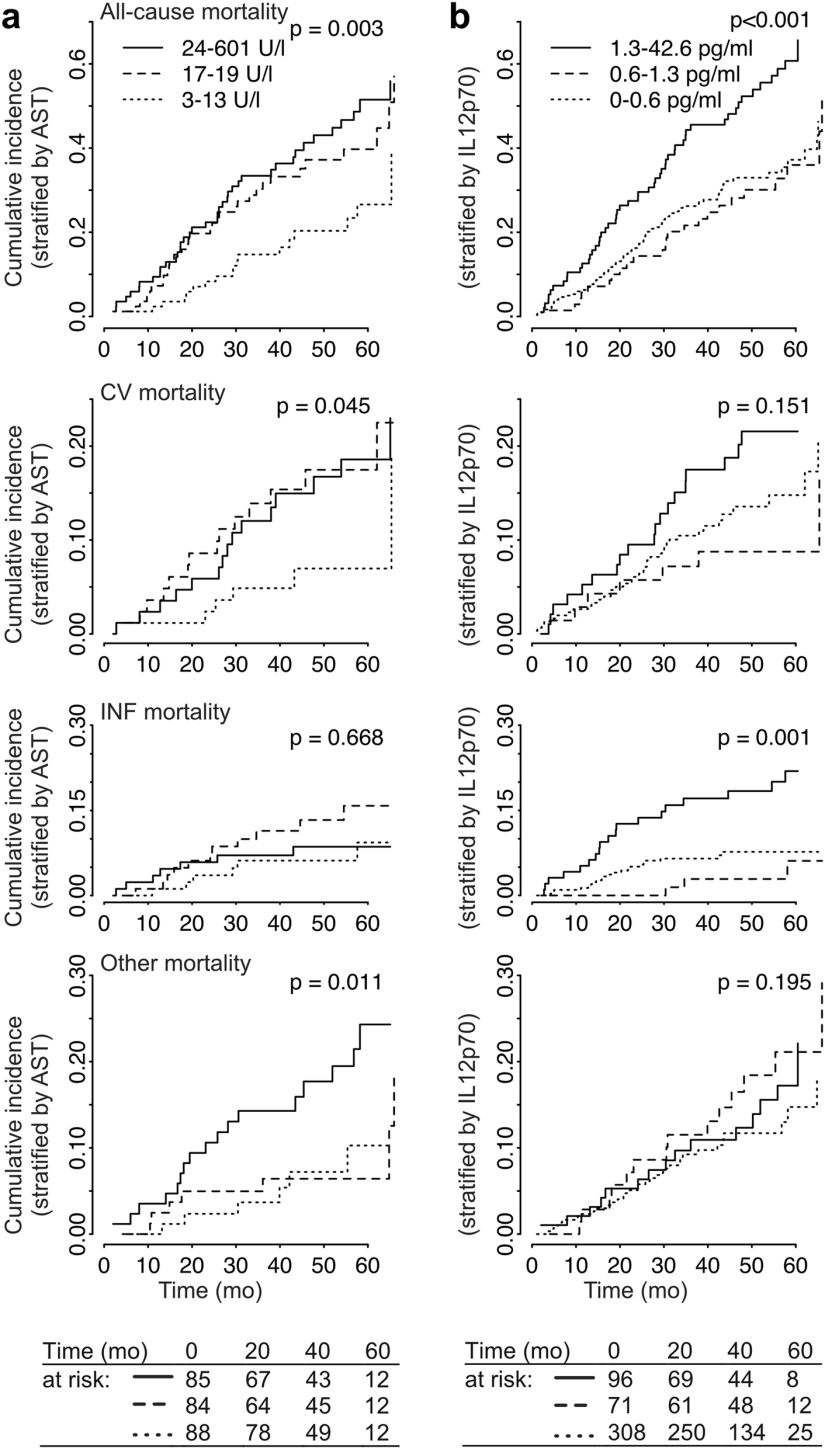
Cumulative incidence plots for all-cause, cardiovascular (CV), infection-associated mortality and other mortality causes. Patients were stratified by AST levels (**a**, patients within top, middle and lowest quintile thresholds) and IL-12p70 levels (**b**, below detection limit, detected but not in top quintile, top quintile threshold). The analysis was performed on the total cohort with non-missing values for the respective variables. P-values were calculated using log-rank test for trend.

**Figure 4:**
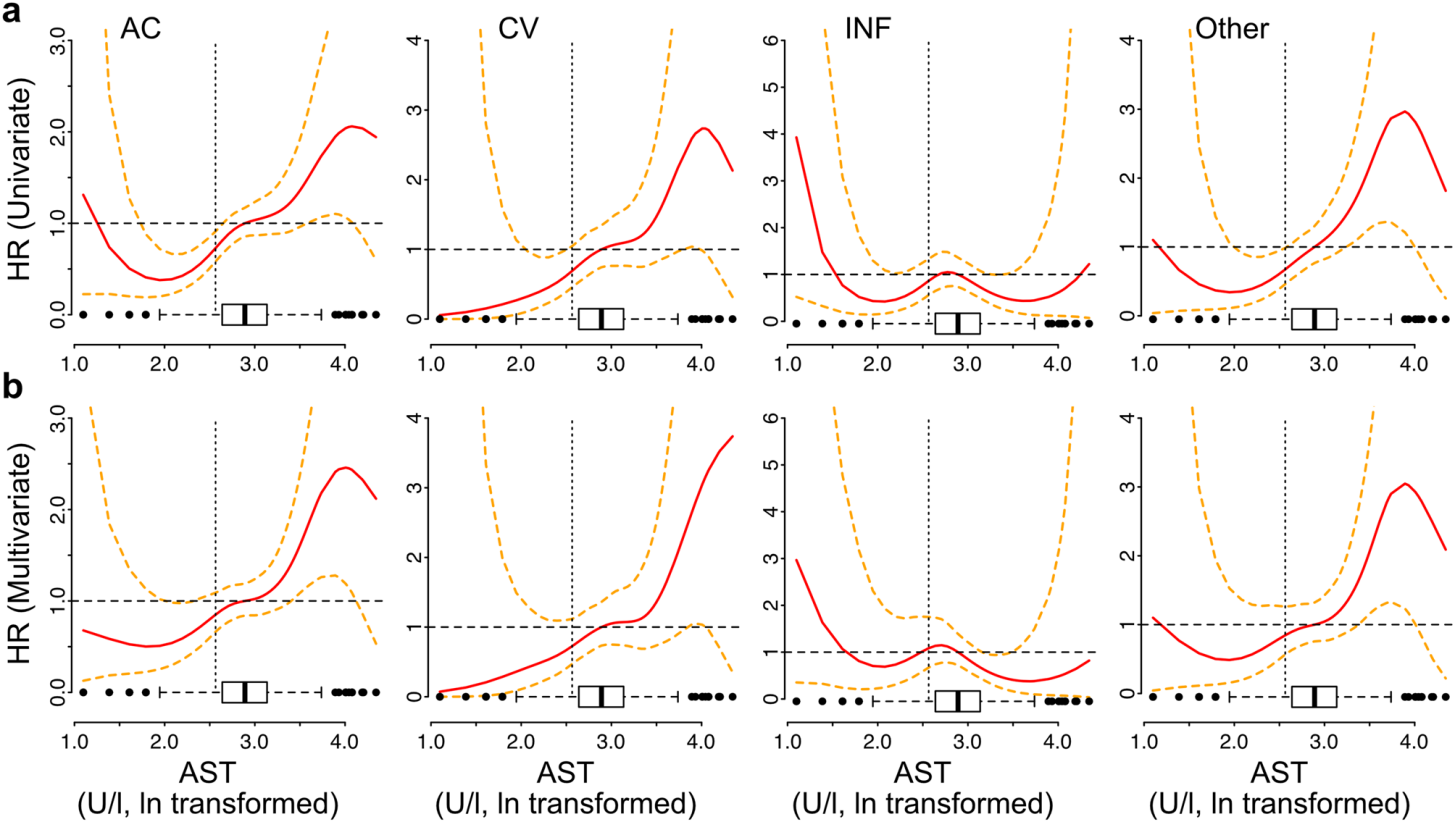
AST spline functions. Spline functions to ln transformed serum AST values were fit in a Cox regression for all-cause, cardiovascular (CV), infection-associated (INF), and other mortality causes. Horizontal boxplots represent the distribution of ln-transformed AST values in ISAR patients. Red lines represent the estimated (cause-specific) HR (patients with median AST as reference) and the dashed lines represent the 95% confidence interval of HR at a given AST level. The models were univariate (**a**) or were adjusted for other relevant predictors in a multivariable model (**b**) and were fit to the total cohort with non-missing values for the respective variables. Multivariable model predictors are described in “Methods” section. Vertical dashed line represents the cutoff value for the lowest AST quintile.

**Figure 5:**
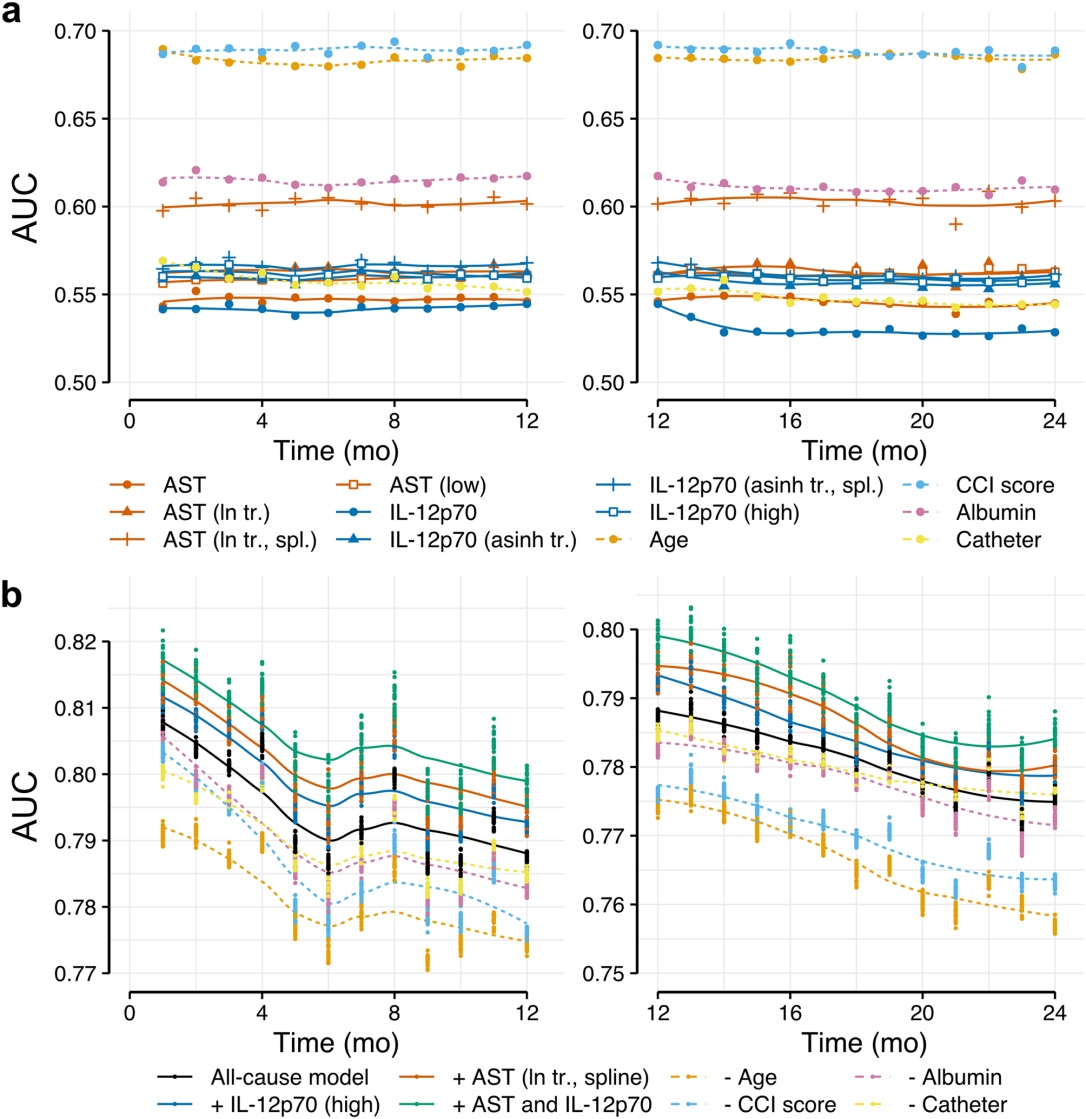
Time-dependent area under the curve (AUC) calculated using bootstrapping. (**a**) Univariate analyses comparing individual variables and transformations. For AST ln transformation was applied. IL-12p70 values were transformed using areasinus hyperbolicus (asinh) due to a large number of patients with measurements below the detection limit. Dashed lines represent established predictors for comparison (as indicated in the color legend). (**b**) Multivariable analysis was fitted using known predictors as described in “Methods” (“All-cause model”, black curve). Solid lines represent the addition of AST (ln-transformed, fitted by a spline) and/or IL-12p70 (high group) to the all-cause model. Dashed lines represent the all-cause model after removal of the indicated known predictors. Dots in (**b**) represent AUC values for individual imputed datasets for each model (see “Methods”). Lines in (**a**) and (**b**) represent smoothed conditional means. Bootstrapping was performed on the total cohort with non-missing values for (**a**) and on the total cohort (n = 475) with missing values imputes as described in “Methods” section for (**b**). Tr., transformation; spl., spline fit; Catheter, use of catheter for dialysis.

Low serum AST levels were associated with decreased all-cause mortality (hazard ratio (HR), 0.31; 95% confidence interval (CI), 0.10–0.98, p = 0.046, in the confirmation subcohort, Table 2). In this “low” group, AST levels were in the range of 3–13 U/l and therefore on the lower end or even below the reference range for the normal population (10–50 U/l for males and 10–35 U/l for females). When analyzing mortality binned by AST subgroup in the total study population, this protective effect could be observed for all-cause and cardiovascular mortality, while increased levels of AST (top quintile) were associated with increased mortality for all-cause and “other” mortality causes (Fig. 3a). Modelling mortality using spline fitting on log transformed AST values significantly outperformed a regular Cox model (p = 0.038 for all-cause mortality in a likelihood ratio test on the total population with non-missing AST-values, n = 377, one patient was excluded due to strongly increased AST and ALT as discussed in “Methods”). We additionally observed a numerically improved predictive performance for a spline fitted model as further discussed below (Fig. 5a). In univariate and multivariable Cox regression incorporating spline transformations a similar association as described above for both a protective effect of lower than average AST as well as a detrimental effect of increased AST levels for all-cause mortality as well as cause-specific hazard for cardiovascular mortality and other causes were identified (Fig. 4). Of note, unknown mortality causes were also classified as “other” causes, we therefore expect some degree of overlap with cardiovascular mortality within this group due to e.g. sudden cardiac death.

Interestingly, despite a strong correlation of serum AST with ALT levels (Pearson coefficient: 0.62, p < 0.001), ALT levels were not significantly associated with all-cause or cardiovascular mortality (not shown) and did not show a similar protective effect of lower values when spline transformations were considered (Figure S2).

### IL12p70 as a predictor of infection-associated mortality in dialysis patients

High IL-12p70 was one of the most stable predictors associated with mortality due to infection (Fig. 2c), showing a significant association also in the confirmation cohort (infection-associated HR 3.12, 95% CI 1.16–8.41, p = 0.024, Table 2). Criteria for stability selection were missed by a small margin for all-cause mortality (Fig. 2a). With the applied assay for IL-12p70, the majority of patients showed an undetectable level of this interleukin (Figure S3). When analyzing mortality binned by IL-12p70 expression (undetectable, weakly elevated, top quintile) in the total study population, we could observe a selective increase in mortality in the top quintile without according tendency in the weakly elevated group (Fig. 3b).

### Added value of AST and IL-12p70 for mortality prediction

Time-dependent receiver-operating characteristics (ROC) analysis for univariate Cox models showed highest areas und the ROC curve (AUC) for AST after logarithmic transformation and spline fitting (Fig. 5a). Using the predefined threshold (top quintile) for IL-12p70 had a similar AUC for all-cause mortality as using transformed full data and when fitting a spline (Fig. 5a). Fitting a spline to transformed IL-12p70 data also did not show a significant improvement in a likelihood ratio test (p = 0.209 for all-cause and p = 0.137 for infection-associated mortality, n = 475).

The AUCs for these single predictors were comparable to those of other established predictors such as serum albumin and the use of a catheter for dialysis, though not as high as some of the top predictors for mortality, age and adapted comorbidity score (Fig. 5a).

Analysis of multivariable models showed an additive effect of the new predictors on our previously established model^5^ (Fig. 5b, see “Methods” for included predictors). In fact, inclusion of both AST and IL-12p70 (green line in Fig. 5b) was superior to albumin or catheter use and had a similar additive value on AUC as the strong predictors age and adapted CCI within the first year of follow up (orange and blue dashed lines in Fig. 4b left panel).

## Discussion

Here, we applied techniques of statistical learning to analyze a cohort study of dialysis patients. Although similar techniques have been previously applied in this population in order to improve mortality prediction^18–20^, our approach differs in that our main goal was not to derive an overall prediction model or to address a specific hypothesis, but rather to find novel promising candidates as predictors in a setting with a large amount of available characteristics for a relatively small study population. Many of the well-known and established prognostic factors for mortality were recovered, while a relatively modest number of 317 patients (with 112 events during follow-up) were used for selection among ≈ 110 predictors. We were then able to confirm significant associations in the withheld smaller confirmation group for most of the factors obtained after stability selection using univariate Cox regression. These findings underline the overall validity of the selection process in order to identify important mortality predictors. A notable observation was thereby the identification of a previously unknown association of IL-12p70 with infection-associated mortality.

IL-12p70 is the active heterodimer form of IL-12, which consists of the IL-12p35 and IL-12p40 subunits. While IL-12p35 is thought to be specific for IL-12, IL-12p40 also associates with IL-23p19 to form active IL-23. IL-12 activity has been implicated in the immunologic response to pathogens and tumor cells, as well as in autoimmune disease^21,22^. Levels of IL-12p70 are known to be relatively low in the serum of healthy individuals^23^ and were below the detection limit for our assay in the majority of the study population. Contradictory reports concerning IL-12 activity exist for dialysis patients. One study, published shortly after the discovery of this cytokine, observed an increased level of IL-12 in dialysis patients compared to healthy controls^24^, a finding that could possibly be explained by increased IL-12 release from peripheral blood mononuclear cells (PBMCs) as an inflammatory response to the dialysis membrane^25^. This study also reported, contrary to our observation, that increased IL-12 levels were associated with improved survival in dialysis patients^24^. However, in a more recent report assessing specifically the levels of IL-12p40 and IL-12p70, a significant increase of IL-12p40 was observed, while no increase was reported for IL-12p70 in dialysis patients vs. healthy controls^26^. The discrepancy between our finding and those of Kimmel et al.^24^ could therefore be explained by the specificity for IL-12p70 in our assay. Alternatively, since we categorized data and compared individuals with 20% highest levels to others, the effects of IL-12 might be non-linear, in that they might be protective up to a certain level and detrimental or indicative of an additional underlying disease (e.g. autoimmune) above that level. Further studies employing more sensitive IL-12p70 and IL-12p40 measurements would be of interest to solve this question. Having high levels of IL-12p70 was one of the most stable predictors for increased infection-associated mortality in our derivation cohort. Although a causal relationship cannot be established by the applied approach, this close correlation suggests that it may be a causal factor or that it may be closely linked to a process causing mortality in hemodialysis patients. Further studies into the underlying pathophysiology would be highly interesting to elucidate this relationship and clarify if modulation of IL-12 activity may serve as a therapeutic intervention.

Another stable predictor which was obtained in our analysis is AST and we further looked into this association because until now it has received little attention in dialysis patients. AST is often used in clinical practice to assess liver damage, however unlike ALT it is less specific and is also elevated in other conditions, such as myocardial infarction or skeletal muscle damage^27,28^. Levels of AST have been shown to be predictive of both all-cause as well as specifically cardiovascular mortality (potentially due to an association with the metabolic syndrome) in the general population^29–32^. While on average a decreased AST activity in dialysis patients compared to the general population was observed long ago^33,34^, we could only find one previous study analyzing its association with mortality in dialysis patients^17^ (in this case with all-cause mortality). Our results confirm these findings and suggest a potential association with cardiovascular disease in dialysis patients. An association with liver disease may explain an increased mortality due to "other" causes in patients with high AST values, similar as was observed for increased ALT values (Figure S2). The finding that ALT levels do not show a similar association with cardiovascular mortality, however, suggests that there may be an alternative relevant pathomechanism or a source of AST elevation outside the liver (such as the heart) which is worthy of further investigation. Although our study is not sufficiently powered to further elucidate the pathophysiology behind AST variation (e.g. only 5% of participants had a known liver disease based on medical records) our results underline the prognostic value of AST and support the previously expressed need for different reference ranges in hemodialysis patients^35^.

When assessing the additional value of AST and IL-12p70 for the prediction of mortality in dialysis patients using time-dependent ROC analysis, we found an additive value of these predictors. Although in the multivariable model the numeric gain was relatively modest, it has to be compared to other established variables, because the model already incorporated many important predictors. We observed a similar effect of AST and IL-12p70 as e.g. for known independent predictors albumin and catheter use for dialysis. The added effect of these two predictors together was similar in the first year to some of the strongest predictors: age and adapted CCI score.

One of the limitations of the applied penalized Cox regression is that it assumes linear associations with the outcome (as does regular Cox regression). This also means that single high-leverage points, which can represent false measurements or extraordinary conditions, may have a strong effect on selection (or omission) of variables. Here we addressed these issues by “dummy coding” quantitative measurements to nominal variables which represent top or lowest quintiles for specific values. Although such thresholding may influence the selection due to loss of information, we in fact observed that most known ordinal/continuous predictors for mortality in dialysis patients passed our selection criteria (i.e. hsCRP, hemoglobin, creatinine, triglycerides, IL-6, YKL-40, AST). However, one notable exception is serum albumin, which was not retained despite a well-documented correlation in the literature. Decreased albumin levels are both observed as part of malnutrition as well as inflammation (anti-acute phase protein), also summarized as dialysis-associated inflammation-malnutrition complex^36^. There is also a known negative correlation between IL-6 and albumin^37,38^. Other retained predictors are also associated with inflammation-malnutrition in ESRD patients, such as hsCRP, creatinine or lipids^36^. It is therefore likely that, next to the thresholding approach, such predictors have additionally diminished the explanatory value of albumin for mortality prediction, as has been reported previously^39^. A similar collinearity of hsCRP with IL-6^40,41^ may have reduced the predictive value of the former in the case of infection-associated mortality, yet it was selected as a predictor for all-cause mortality where more events were available and potentially due to its described additional role as a predictor of malnutrition^42^.

We strongly assume that including more patients in the derivation cohort would further improve factor selection, while in our study this dataset was relatively small (i.e. 317 patients). Having a larger cohort would also allow to include variable interactions in order to identify subgroup-specific effects, something that was not accounted for in our approach. Further limitations of our study are missing values for some of the variables and unknown cause of death for some of the patients. We expect that a relevant portion of missing values, as these were set to the reference level (see “Methods”), will reduce the selection stability of a variable and therefore typically lead to a more conservative selection. An additional conceptual limitation is that only the variables at baseline could be used in the current analysis. Yet, the evolution of biomarkers over time in dialysis patients and the course of chronic kidney disease may provide additional important insights and improve predictive performance.

In summary, we successfully applied techniques of statistical learning as an exploratory approach to high-dimensional data from a cohort of dialysis patients. Despite the relatively modest number of patients, we thereby could retrieve most of the established risk markers and additionally identify promising candidates for novel predictors. The described techniques can be considered complementary, yet not as competitors to widely-applied and state of the art hypothesis-driven approaches. Similar techniques, as they become more accessible, may gain importance in routine exploratory study analysis in order to gain additional insights into the underlying risk-determining factors.

## Methods

### Subjects/study population and data collection

The clinical trial was registered under ClinicalTrials.gov identifier: NCT01152892. For detailed information please refer to the study protocol^12^. In brief, we included stable HD patients who were at least 18 years of age, had an HD vintage of at least 90 days, and provided written and informed consent. Malignant disease with a life expectancy shorter than 2 years had been the only relevant exclusion criterion. The present study included 475 patients, for whom sufficient unthawed sera were available for cytokine quantification. The study was approved by the ethics committees of the Klinikum rechts der Isar, Technical University Munich, and by the Bavarian State Board of Physicians. It was performed in accordance with the Declaration of Helsinki.

Age, patient characteristics, dialysis modalities, comorbidities and medication were assessed from medical records. Comorbidities were recorded at inclusion and updated during the 2 years follow up as previously described^5,43^. An adapted CCI was calculated according to Liu et. al.^13^ (version without ESRD cause).

Cause of death was attributed to the following domains: cardiovascular, infection associated and other (or unknown) of which the cardiovascular domain had been prespecified in the study protocol^12^. For further details also see Lorenz et al.^5^ Medical records and interviews of attending physicians, and relatives were used by the ISAR study physician board to ascertain death and assigned each case an underlying cause of death. When no agreement on cause of death could be reached the cause of death was defined as unknown (n = 28).

### Serumbiochemistry and functional studies

Serum was collected prior to a midweek dialysis session at inclusion and after the exclusion of active infection. Patients that had undergone surgery within the past 2 weeks were included at a later timepoint. Serum was centrifuged after 30 min of resting at room temperature, aliquoted and stored at – 80 °C. Routine laboratory analyses were assessed by International Organization for Standardization–certified laboratories. YKL-40 levels were measured in duplicates using a commercial enzyme-linked immunosorbent assay (ELSIA) kit (R&D Systems, Inc., Minneapolis, MN). The remaining inflammatory mediators were assessed using BD Flex-sets (Becton Dickinson, Heidelberg, Germany) on a FACS Canto II (Becton Dickinson) and BD Diva software (Becton Dickinson) according to manufacturer’s instructions. Theoretical limits of detection were indicated by the manufacturer between 0.6 and 1.6 pg/mL.

Ambulatory 24-h blood pressure (BP) and pulse wave velocity (PWV) measurements were carried out as previously described^44^ using a Mobil-O-Graph 24 h-PWA (I.E.M. GmbH, Stolberg, Germany). Retinal vessel analysis was performed using Static Vessel Analyzer (Imedos Systems UG, Jena, Germany). Fundus images of one eye were gathered and classified into four categories according to quality. Two images with the highest qualitative category were analyzed and means of central retinal arteriolar (CRAE) and central retinal venular equivalent (CRVE) as well as arteriolar-to-venular diameter ratio (AVR) were calculated as previously described^45^.

### Statistical analyses

Statistical analysis was performed in R version 3.6.3^46^.

In order to be able to test the selected predictors on patients whose data was not used for variable selection, we split the cohort into a derivation and confirmation datasets. To this end we performed 1000 random splits in the ratio of 2/3 to 1/3 and calculated for each split the p-value for difference in the matching variables (age, adapted CCI, gender, IL-6 and mortality as indicated in Table 1) using Chi-squared test for nominal and Mann–Whitney test for ordinal or continuous variables. We then selected the split where the two groups were most likely similar (based on where the lowest p-value of the tests was the highest).

For stable variable selection we applied elastic net regularized regression on the derivation subcohort on all predictors listed in Table S1 simultaneously. In order to address possible non-linearity, ordinal and continuous variables (with the exception of age for which a linear relationship can be expected and which was expressed in decades) were dummy coded as “high” and “low” group by assigning for each patient whether he falls into the top or bottom quintile for the specific variable. Ties were assigned randomly. Values not falling into top or bottom quintile and missing values were assigned 0 in both dummy variables. Missing values in nominal variables were also assigned to the reference category (meaning absence of a potential risk factor).

In a first step we performed elastic net^10^ Cox regression separately for all-cause mortality, cardiovascular and infection-associated mortality with 20-fold cross-validation using 15 equally spaced *alpha* values in the range of 0 to 1 (here *alpha* = 0 equals ridge regression and *alpha* = 1 equals lasso regression). We selected for each *alpha* the *lambda* with the lowest cross-validation error (Figure S1a). We then selected for each regression the non-zero *alpha* value with the lowest cross validation error (vertical dashed lines in Figure S1a). *Alpha* of zero would not result in any variable selection (as it equals ridge regression) and was therefore not chosen. Next, we used the chosen *alpha* values in a stability selection approach as described by Meinshausen et Bühlmann^11^ and implemented in R by Sill et al.^47^ For this preselection approach as a means of hypothesis generation we used 100 subsamples of the derivation cohort, a “weakness” of 0.8 and a (maximal) per-comparison error rate of 10%. All variables passing the selection criteria are reported in Table 2.

For spline fitting we used penalized splines^48,49^ with four degrees of freedom. For the analysis in Fig. 4 and Figure S2 one outlier with AST of > 600 U/l and ALT > 900 U/l (suggestive of acute hepatopathy) was removed.

The multivariable Cox model for all-cause mortality (Fig. 4b, Figure S2 and Fig. 5b) contained the following predictors (as described previously^5^): age, gender, BMI, adapted CCI, history of MI, rheumatic disease, laboratory parameters (phosphate, calcium, albumin), Kt/V, HD (not hemodiafiltration), usage of central venous catheter for dialysis, oral anticoagulation, IL-6 (ln-transformed) and YKL-40 (ln-transformed). The multivariable model for cardiovascular mortality contained: age, gender, BMI, adapted CCI, history of MI, rheumatic disease, usage of central venous catheter for dialysis, ultrafiltration per kilogram of dry weight, IL-6 (ln-transformed) and YKL-40 (ln-transformed). The model for infection-associated mortality contained age, gender, BMI, adapted CCI, history of MI, Albumin, usage of central venous catheter for dialysis, hsCRP (ln-transformed) and IL-6 (ln-transformed). For other mortality causes we used a minimal model which included: age, gender, BMI, adapted CCI and IL-6 (ln-transformed).

Time-dependent ROC analysis for Cox regression was performed by first fitting uni- (Fig. 5a) or multivariable (Fig. 5b) models to a bootstrap sample of the total study population and then calculating incident/dynamic AUCs^50^ on the out of bootstrap sample. The bootstrap procedure was performed 1000 times and the resulting AUCs were averaged per month. For multivariable analysis missing values were randomly sampled from the available values for the respective variable and a total of 25 thus imputed datasets were generated. AUCs for each of these datasets were calculated as described above (these are presented as dots in Fig. 5b).

## Supplementary Information

Supplementary Information.
