# Supplementary material for: Application of regularized regression to identify novel predictors of mortality in a cohort of hemodialysis patients
Source: Sci Rep. 2021 Apr 29;11:9287. doi: 10.1038/s41598-021-88655-0 (PMC8085040; doi:10.1038/s41598-021-88655-0)
Supplement: Supplementary file 1 — Supplementary Information. [file 41598_2021_88655_MOESM1_ESM.pdf]

Supplementary information

Application of regularized regression to identify novel predictors of mortality in a cohort of hemodialysis patients

Stanislas Werfel

Georg Lorenz

Bernhard Haller

Roman Günthner

Julia Matschkal

Matthias C. Braunisch

Carolin Schaller

Peter Gundel

Stephan Kemmner

Salim S. Hayek

Christian Nusshag

Jochen Reiser

Philipp Moog

Uwe Heemann

Christoph Schmaderer

Table S1. List of all variables in the selection approach and cohort characteristics.

Variable	Total cohort	n-missing	Quintile cutoffs	Derivation cohort	Confirmation cohort
n (%total)	475 (100%)			317 (67%)	158 (33%)
BMI (kg/m²)	25 [23-29]	0	(<=22 / >=30)	25 [23-29]	25 [22-29]
Dialysis vintage (mo)	43 [22-80]	0	(<=18 / >=92)	48 [24-83]	37 [17-73]
Cardiovscular disease (CVD)	174 (37%)	0		112 (35%)	62 (39%)
H.o. MI	95 (20%)	0		58 (18%)	37 (23%)
LV hypertrophy	146 (31%)	0		109 (34%)	37 (23%)
Heart failure (HF)	90 (19%)	0		55 (17%)	35 (22%)
Atherosclerosis (non CHD)	163 (34%)	0		116 (37%)	47 (30%)
Arterial hypertension (AHT)	443 (93%)	0		300 (95%)	143 (91%)
Other cardiac disease	35 (7%)	0		19 (6%)	16 (10%)
Atrial fibrillation (AF)	109 (23%)	0		67 (21%)	42 (27%)
Pace maker	39 (8%)	4		30 (10%)	9 (6%)
HRD (non AF)	82 (17%)	0		63 (20%)	19 (12%)
Cardiac valvular disease	110 (23%)	0		74 (23%)	36 (23%)
Peripheral artery disease (PAD)	113 (24%)	0		82 (26%)	31 (20%)
H.o. amputation due to PAD	35 (7%)	0		25 (8%)	10 (6%)
Pulmonary hypertension	35 (7%)	0		22 (7%)	13 (8%)
Central vascular disease	78 (16%)	0		59 (19%)	19 (12%)
COPD	52 (11%)	0		32 (10%)	20 (13%)
Lung fibrosis	2 (0%)	0		2 (1%)	0 (0%)
Infectious disease (HIV, Hep. B/C)	50 (11%)	0		34 (11%)	16 (10%)
H.o. neoplasia	100 (21%)	0		58 (18%)	42 (27%)
Vasculitis	23 (5%)	0		15 (5%)	8 (5%)
Gastrointestinal (GI) disease	179 (38%)	0		126 (40%)	53 (34%)
Liver disease	25 (5%)	0		16 (5%)	9 (6%)
Rheumatologic disease	23 (5%)	0		18 (6%)	5 (3%)
Depressive disorder	35 (7%)	0		19 (6%)	16 (10%)
Dementia	10 (2%)	0		9 (3%)	1 (1%)
H.o. drug abuse	36 (8%)	0		28 (9%)	8 (5%)
Diabetes mellitus (DM)	192 (40%)	0		131 (41%)	61 (39%)
Hypercholesterolemia	295 (62%)	0		197 (62%)	98 (62%)
Smoking	106 (22%)	3		72 (23%)	34 (22%)
ESRD D/T DM	93 (20%)	0		65 (21%)	28 (18%)
ESRD D/T GN	92 (19%)	0		54 (17%)	38 (24%)
ESRD D/T AHT	80 (17%)	0		59 (19%)	21 (13%)
ESRD D/T inherited disease	47 (10%)	0		29 (9%)	18 (11%)
ESRD D/T other causes	163 (34%)	0		110 (35%)	53 (34%)
ACE inhibitor	175 (37%)	0		111 (35%)	64 (41%)
AT1-blocker	106 (22%)	0		73 (23%)	33 (21%)
Calcium channel blocker	187 (39%)	0		126 (40%)	61 (39%)
Vasodilator	58 (12%)	0		39 (12%)	19 (12%)
Beta-blocker	319 (67%)	0		215 (68%)	104 (66%)
Diuretics	293 (62%)	0		184 (58%)	109 (69%)
Other BP medication	82 (17%)	0		60 (19%)	22 (14%)
Statin	186 (39%)	0		127 (40%)	59 (37%)
Oral anticoagulation	77 (16%)	0		55 (17%)	22 (14%)
Immunosuppression	41 (9%)	0		24 (8%)	17 (11%)
Kt/V	1.46 [1.23-1.66]	0	(<=1.20 / >=1.75)	1.47 [1.21-1.70]	1.44 [1.25-1.60]
Haemodialysis(=1)/Haemodiafiltration(=0)	407 (86%)	0		271 (85%)	136 (86%)
Dialysis catheter	33 (7%)	0		25 (8%)	8 (5%)
Dialysis time (h)	4.3 [4.0-4.5]	0	(<=4.0 / >=4.8)	4.3 [4.0-4.5]	4.2 [4.0-4.5]
Ultrafiltration (ml/kg dry weight)	30 [21-39]	0	(<=17 / >=42)	29 [21-40]	31 [21-39]
Total removed volume (l)	1.8 [1.0-2.6]	6	(<=0.8 / >=2.8)	1.8 [1.0-2.5]	1.8 [1.0-2.7]
Heparin (dialysis)	389 (82%)	0		255 (80%)	134 (85%)
LMW heparin (dialysis)	72 (15%)	0		55 (17%)	17 (11%)
Other ac (dialysis)	14 (3%)	0		7 (2%)	7 (4%)
Serum creatinine (mg/dl)	8.3 [6.4-10.3]	0	(<=6.1 / >=10.8)	8.5 [6.8-10.4]	7.9 [5.9-10.2]
Blood Urea Nitrogen (mg/dl)	61.2 [50.2-71.5]	0	(<=48.4 / >=73.9)	61.6 [50.4-70.5]	61.1 [50.0-72.7]
Serum hsCRP (mg/l)	0.46 [0.20-0.97]	12	(<=0.15 / >=1.15)	0.47 [0.21-0.93]	0.42 [0.15-1.03]
Serum iron (µg/dl)	64.0 [48.0-81.2]	51	(<=45.0 / >=88.5)	64.0 [48.0-79.0]	66.0 [51.0-87.0]
Transferrin saturation (%)	25 [19-33]	53	(<=18 / >=35)	25 [19-33]	26 [19-34]
Serum ferritin (µg/l)	612 [359-967]	39	(<=305 / >=1056)	590 [358-941]	661 [374-1028]
Serum transferrin (mg/dl)	176 [152-200]	59	(<=149 / >=207)	176 [150-199]	176 [154-204]

Variable	Total cohort	n-missing	Quintile cutoffs	Derivation cohort	Confirmation cohort
Serum sodium (mmol/l)	139 [137-141]	16	(<=136 / >=141)	139 [137-141]	138 [136-140]
Serum potassium (mmol/l)	5.2 [4.7-5.8]	15	(<=4.6 / >=6.0)	5.3 [4.7-5.8]	5.2 [4.7-5.8]
Serum calcium (total, mmol/l)	2.27 [2.16-2.38]	2	(<=2.13 / >=2.41)	2.27 [2.14-2.38]	2.28 [2.18-2.40]
Serum phosphate (mmol/l)	1.6 [1.4-2.0]	0	(<=1.3 / >=2.1)	1.7 [1.4-2.0]	1.6 [1.3-2.0]
Serum Ca*P (mmol²/l²)	3.8 [3.1-4.7]	3	(<=3.0 / >=4.8)	3.8 [3.1-4.7]	3.8 [3.1-4.7]
Serum AST (U/l)	18 [14-23]	97	(<=13 / >=24)	18 [14-22]	18 [14-24]
Serum ALT (U/l)	17 [13-24]	37	(<=12 / >=26)	18 [13-24]	17 [13-24]
Serum albumin (g/dl)	4.0 [3.8-4.2]	0	(<=3.7 / >=4.3)	4.0 [3.8-4.2]	4.1 [3.7-4.3]
Hb (g/dl)	11.7 [11.1-12.4]	0	(<=10.9 / >=12.6)	11.7 [11.0-12.4]	11.7 [11.2-12.4]
HCT (%)	35.0 [33.0-37.8]	5	(<=32.0 / >=38.4)	35.0 [33.0-37.8]	35.0 [33.1-38.0]
Leukocytes (10^9/l)	6.70 [5.50-8.07]	0	(<=5.20 / >=8.41)	6.80 [5.50-8.20]	6.60 [5.40-7.90]
Platelets (10^9/l)	202.0 [165.0-247.0]	1	(<=160.0 / >=259.0)	198.5 [164.0-246.0]	206.0 [168.2-250.0]
Serum cholesterol (mg/dl)	172 [144-202]	96	(<=138 / >=210)	172 [144-201]	178 [143-202]
Serum HDL (mg/dl)	43 [36-53]	110	(<=35 / >=57)	43 [36-53]	44 [38-53]
Serum LDL (mg/dl)	107 [78-130]	110	(<=73 / >=138)	106 [79-129]	108 [77-132]
Serum triglycerides (mg/dl)	146 [108-221]	142	(<=96 / >=242)	146 [100-221]	154 [114-210]
Intact PTH (pg/ml)	225.2 [110.2-394.5]	27	(<=94.6 / >=440.5)	227.4 [106.4-394.6]	222.4 [119.5-394.2]
Serum T50 (min)	2.43 [1.97-2.85]	6	(<=1.90 / >=2.98)	2.45 [1.98-2.88]	2.36 [1.94-2.82]
Serum IL-1b (pg/ml)	0.00 [0.00-2.74]	0	(<=0.00 / >=3.19)	0.00 [0.00-2.49]	0.00 [0.00-2.98]
Serum IL-2 (fg/ml)	42.5 [0.0-101.6]	0	(<=0.0 / >=120.7)	40.3 [0.0-94.5]	46.7 [0.0-119.2]
Serum IL-4 (fg/ml)	23.3 [0.0-84.7]	0	(<=0.0 / >=101.6)	18.3 [0.0-81.5]	36.4 [0.0-95.4]
Serum IL-6 (pg/ml)	9.44 [5.62-16.14]	0	(<=4.74 / >=18.15)	9.21 [5.60-15.61]	10.22 [5.71-16.57]
Serum IL-7 (pg/ml)	7.10 [0.00-11.10]	4	(<=0.00 / >=12.59)	7.01 [0.00-10.49]	7.31 [0.00-11.86]
Serum IL-10 (fg/ml)	654 [360-1447]	0	(<=309 / >=1668)	743 [372-1463]	569 [331-1328]
Serum IL-11 (pg/ml)	0.28 [0.00-11.10]	4	(<=-0.04 / >=14.67)	0.22 [0.00-10.35]	0.45 [0.00-15.11]
Serum IL-12p70 (pg/ml)	0.00 [0.00-0.97]	0	(<=0.00 / >=1.28)	0.00 [0.00-0.92]	0.00 [0.00-1.05]
Serum IL-13 (pg/ml)	2.30 [0.00-3.62]	4	(<=0.00 / >=3.95)	2.29 [0.00-3.54]	2.33 [0.00-3.71]
Serum IL-17A (fg/ml)	86.2 [26.3-225.7]	0	(<=14.3 / >=298.3)	81.6 [19.7-193.6]	96.5 [39.8-263.4]
Serum Granzyme B (pg/ml)	1.88 [0.00-9.51]	4	(<=0.00 / >=13.16)	1.91 [0.00-9.23]	1.34 [0.00-11.11]
Serum Fractalkine (pg/ml)	36.51 [23.12-51.34]	4	(<=19.40 / >=55.70)	36.81 [22.96-50.76]	35.02 [24.03-52.70]
Serum GMCSF (pg/ml)	1.80 [0.00-3.96]	4	(<=0.00 / >=4.71)	1.60 [0.00-3.62]	2.03 [0.00-4.14]
Serum IFN-gamma (fg/ml)	59.5 [0.0-260.6]	0	(<=0.0 / >=358.4)	59.0 [0.0-274.8]	60.1 [0.0-237.1]
Serum IP-10 (pg/ml)	199.1 [129.3-355.9]	0	(<=115.3 / >=391.2)	195.3 [127.6-327.4]	209.2 [137.9-389.5]
Serum MCP-1 (pg/ml)	104.8 [62.1-162.9]	0	(<=52.1 / >=183.9)	106.8 [62.5-173.6]	100.5 [58.0-153.9]
Serum MRP (µg/ml)	2.77 [1.84-4.19]	6	(<=1.62 / >=4.64)	2.89 [1.77-4.02]	2.68 [1.88-4.44]
Serum PD-1 (pg/ml)	78.09 [0.00-313.11]	19	(<=0.00 / >=396.67)	95.29 [0.00-337.52]	73.37 [0.00-292.80]
Serum TNFalpha (pg/ml)	2.30 [0.00-4.45]	4	(<=0.00 / >=5.22)	2.24 [0.00-4.57]	2.42 [0.00-4.38]
Serum YKL-40 (pg/ml)	130.7 [85.4-193.0]	0	(<=77.6 / >=229.1)	133.7 [85.4-192.8]	128.8 [85.7-196.1]
Dialysate Sodium (mmol/l)	138 [138-138]	0	(<=138 / >=138)	138 [138-138]	138 [138-138]
Dialysate Calcium (mmol/l)	1.25 [1.25-1.50]	0	(<=1.25 / >=1.50)	1.25 [1.25-1.50]	1.25 [1.25-1.50]
Dialysate bicarbonate (mmol/l)	32 [32-32]	0	(<=32 / >=33)	32 [32-32]	32 [32-32]
Mean 24h systolic blood pressure (mmHg)	123 [113-134]	141	(<=111 / >=136)	123 [114-135]	123 [113-132]
Mean 24h arterial pressure (mmHg)	96 [88-105]	141	(<=86 / >=107)	97 [89-106]	96 [88-104]
Mean 24h diastolic blood pressure (mmHg)	74 [65-81]	141	(<=63 / >=84)	74 [65-82]	73 [66-80]
Mean 24h pulse pressure (mmHg)	49 [41-57]	141	(<=40 / >=59)	50 [42-57]	48 [41-54]
Mean 24h heart rate (/min)	71 [63-79]	141	(<=62 / >=80)	70 [63-78]	72 [66-79]
Mean 24h pulse wave velocity (m/s)	9.7 [7.9-11.2]	141	(<=7.2 / >=11.5)	9.7 [7.9-11.1]	9.8 [8.0-11.4]

Note: age and sex were also included as predictors and are summarized in Table 1 (no missing values).
For ordinal/continuous variables numbers in brackets indicate the median and interquartile range.
Abbreviations: ac, anticoagulation; AF, atrial fibrillation; AHT, arterial hypertension; ALT, alanine transaminase; AST, aspartate transaminase; AVR, retinal measure of arteriole to venule ratio; BMI, body mass index; BP, blood pressure; Ca*P, calciumphosphate product; CHD, coronary heart disease; CRAE, central retinal artery equivalent; CRVE,central retinal vein equivalent; CV, cardiovascular; D/T, due to; ESRD, end-stage renal disease; GI, gastrointestinal; GN, glomerulonephritis; H.o., history of; Hb, hemglobin; HCT, hematocrit; HDL, high density lipoprotein; HRD, heart rhythm disorder; LDL, low density lipoprotein; LMW, low molecular weight; LV, left ventricle; MI, myocardial infarction; n, number of subjects; T50, serum calcification propensity test.

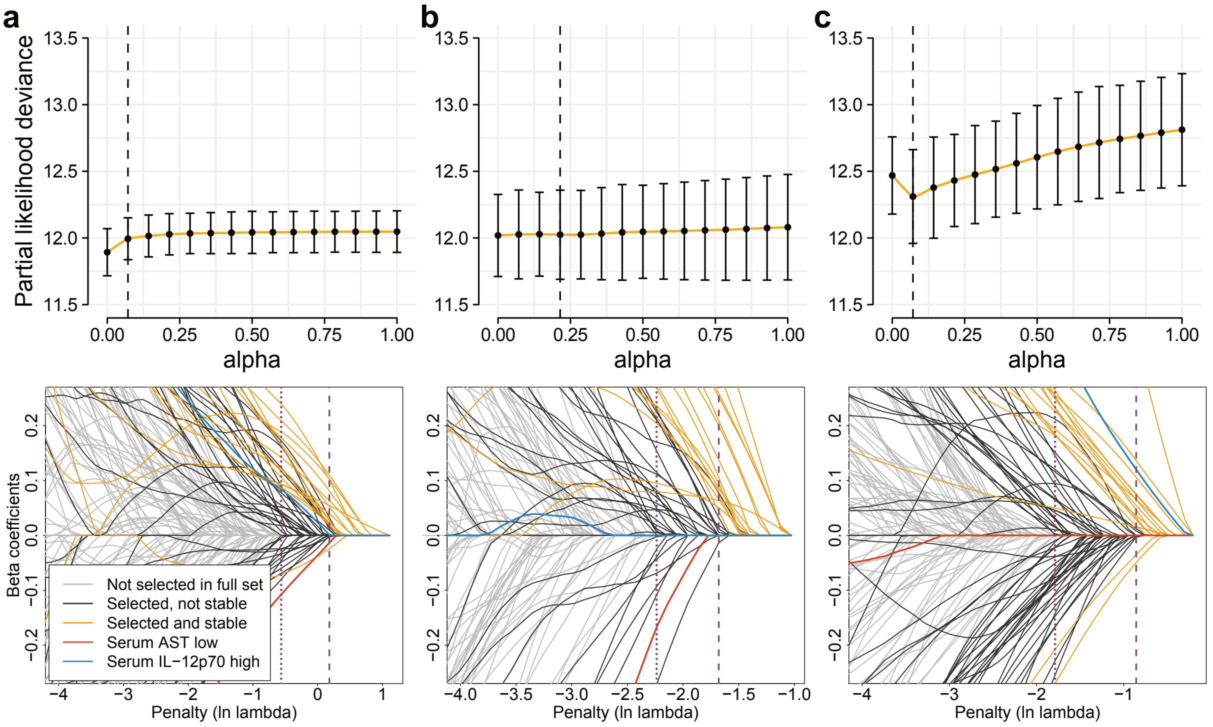

Figure S1. Results of elastic net Cox regression on the derivation dataset of the ISAR cohort for all-cause (a), cardiovascular (b) and infection-associated mortality (c). 20x fold cross validation results for the different *alpha* values are shown in the top panels. Partial likelihood deviance (+- standard deviation) represents prediction error. Vertical dashed lines in the top panels represent the selected alpha values with smallest errors and alpha>0. Bottom panels represent regression coefficients (= ln of regularized HR) for the individual predictors over the penalty parameter (ln of lambda). Each curve represents either a nominal predictor or a group (top/bottom quintile) of an ordinal/continuous predictor as in Figure 2. In the bottom panels the vertical dotted line represents *lambda* at minimal cross-validation error, the vertical dashed line represents lambda chosen by stability selection (which is more stringent). Black lines represent variables which would be additionally selected if no stability analysis was applied (therefore with beta<>0 for a lambda with minimal cross-validation error). The stability selection allows to further reduce the number of potentially relevant variables to those with most stable effects by applying subsampling to the available derivation cohort.

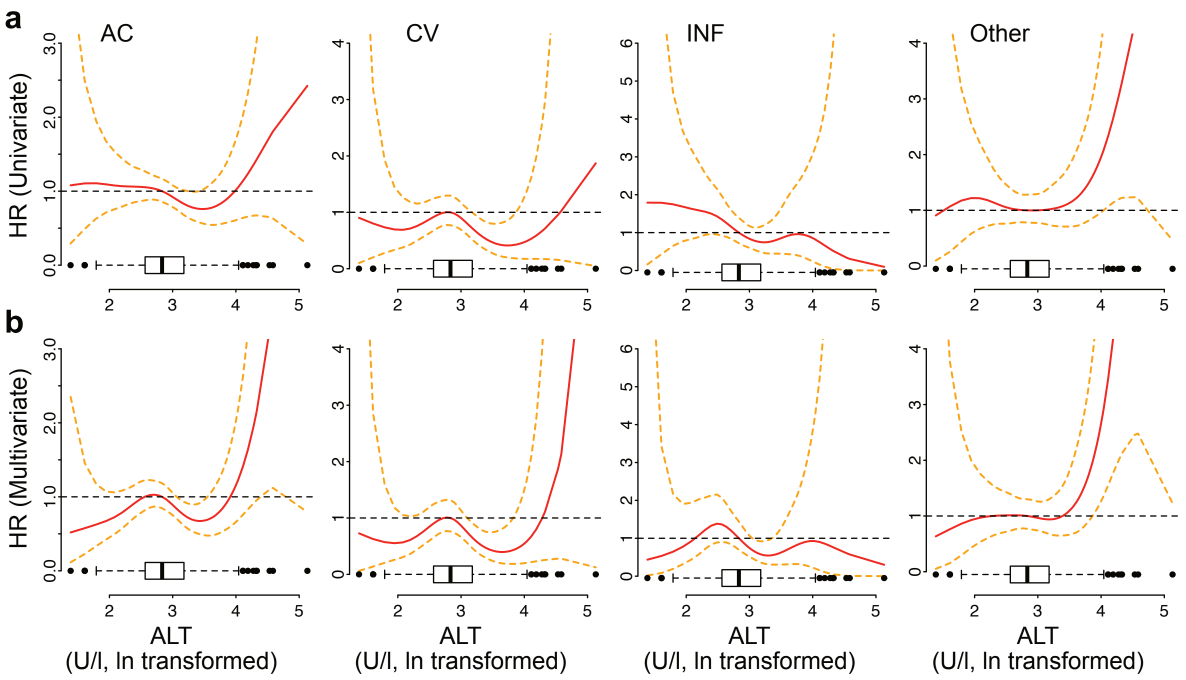

Figure S2. ALT spline functions. Spline functions to ln-transformed serum ALT values were fit in a Cox regression for all-cause, cardiovascular (CV), infection-associated (INF) and other mortality causes, similar as for AST (Figure 4). Horizontal box plots represent the distribution of ALT values in ISAR patients. The models were univariate (a) or were adjusted for other relevant predictors in a multivariable model (b) and were fit to the total cohort with non-missing values for the respective variables. A trend for increased mortality is present for exceptionally high ALT values, however no relevant protective effect of lower values is observed.

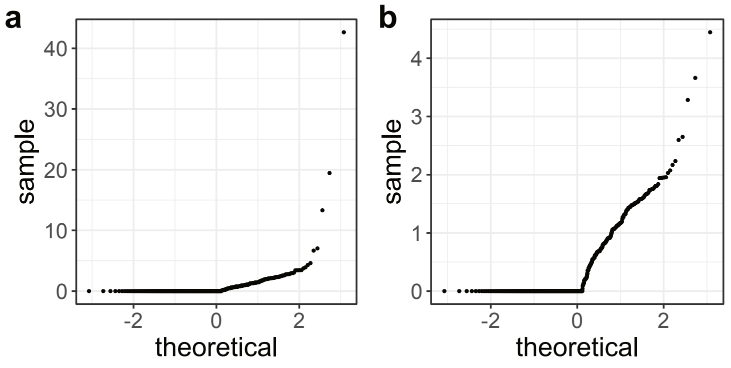

Figure S3. Quantile-quantile plots of the original (a) and asinh (b) transformed IL-12p70 measurements in the ISAR cohort.
